# Kyphoscoliosis with Pulmonary Hypertension

**Published:** 1958-01

**Authors:** T. F. Hewer


### KYPHOSCOLIOSIS WITH PULMONARY HYPERTENSION

(*A Clinical Pathological Conference of the University of Bristol Medical School*)

CHAIRMAN: PROFESSOR T. F. HEWER

*Professor Perry:* This man came to the out-patient department on the 5th March, 1957. He was 48, an industrial chemist working for a firm near Bristol. Three weeks earlier he had noticed rapidly increasing dyspnoea on effort and, for a few days, sub-sternal effort pain.

His previous history is that he had a severe spinal kyphoscoliosis, said to be postural in origin, since childhood. He had visited an orthopaedic surgeon at the age of ten. He had it therefore for at least 38 years. At the age of 22 he had an illness which he was told was lung trouble. He did not remember much about it. At the age of 37 he was an in-patient in the Royal United Hospital, Bath, with a duodenal ulcer which responded well to treatment. Twelve months ago he had a cough for three days and coughed up reddish brown sputum. His doctor sent him to have an X-ray and he was told the X-ray revealed no abnormality, but I do not see how a satisfactory X-ray could have been taken in view of the spinal deformity. During the summer of 1956, he had been playing tennis, family tennis—not Wimbledon Standard. (Laughter.) The family history is that his mother and father are alive and well and in their seventies.

On examination he had very severe kyphoscoliosis. He was cyanosed and had extreme orthopnoea. He could not attempt to lie down. The neck veins were engorged. The liver was enlarged below the costal margin. The heart was in normal rhythm. Blood pressure examination showed a small pulse pressure, 120/90. It was difficult to feel the apex beat or to hear the heart sounds because of the deformity. There were rales over both lung bases. The E.C.G. showed severe right ventricular preponderance and right ventricular strain. He was then in severe congestive heart failure and I arranged for his admission as soon as possible.

He was admitted two days later. The position was much the same, except that he was worse. He was treated initially with digoxin, without response. After two days this was stopped because we wondered if it was the right thing to do. Mersalyl and ammonium chloride were started. He did not respond to treatment. He died ten days after I first saw him and eight days after admission.

The clinical diagnosis was kypho-scoliotic heart disease, one of the first descriptions of which in the English literature was made by Carey Coombs in the *British Journal of Surgery* in 1930. He described four cases which followed the pattern of this man very closely.

Coombs suggested that there might be three possible causal factors of the heart failure:—

(1) The kinking of the thoracic aorta through its clinging too faithfully to the deformed spine (first described by Morgagni).

(2) Inadequate air space in the lungs.

(3) Restriction of the diaphragmatic excursion.

If the first theory is correct it is difficult to explain the marked right ventricular hypertrophy which is always present, but it would fit in with the clinical onset with severe rapidly increasing dyspnoea. The present view is that, in fact, the condition is a form of "cor pulmonale" resulting from the interference with pulmonary function, but the onset of symptoms with dyspnoea remains a puzzle to me. In 1930 the concept of pulmonary hypertension was hardly born but possibly this may develop in these people, perhaps from long continued anoxia and give rise to right ventricular hypertrophy and failure. If this theory is correct it might be a bad thing to treat these cases with digoxin and that was why it was stopped when there was no immediate response.

*Dr. Sutton:* Was there any record of tachycardia?

*Professor Perry:* His heart rate in hospital ranged from 90 to 100 and it was in normal rhythm.

*Dr. Johnson:* The body of this man was well nourished. There was moderately severe oedema of legs and back and severe cyanosis. There was no finger clubbing. There was a severe kyphoscoliosis convex to the right and I have no information to offer as to the cause. There was no evidence of anterior poliomyelitis or of tuberculous caries of the spine. Histological section shows only a little sclerotic buttressing along the concave border. The bodies of the other thoracic vertebrae are normal.

Because of this deformity the lungs were remarkably small and mis-shapen. On section the parenchyma was normal but I was immediately struck by the severe dilatation of the pulmonary artery and its first branches and the moderate atheroma that they showed. There was no disease of the lung parenchyma itself, only of the pulmonary vessels.

A histological section shows normal lung parenchyma, the only change being in the vessels. There is a fair degree of atheromatous thickening of these hypertrophied branches of the pulmonary artery. Apart from this and their small size the lungs were essentially normal.

Because of the kyphoscoliosis the position of the heart was abnormal. The long axis lay transversely across the body. There was kinking of the aorta where it fitted into the concave side of the thoracic curve. There was well-marked right-sided cardiac hypertrophy. Comparison of muscle fibres taken from similar sites shows that those from the right ventricle wall are two or three times the diameter of those from the left and the left ventricle is normal.

Other abnormal findings were a number of small subacute ulcers on the lesser curve of the stomach and one small healed chronic ulcer. There was also a mild degree of cerebral oedema.

I think there are two main problems in this case; one has been indicated to you by Professor Perry. The other is the association of gastric ulceration with right-sided heart failure. The first problem is why severe kyphoscoliosis causes right ventricular hypertrophy and heart failure. To give some indication of the position of this problem I have prepared a table showing conditions associated with pulmonary hypertension abstracted from the last 2,000 post-mortems performed in this Department. In the last 6 years we have had three cases in which there has been kyphoscoliosis with marked right ventricular hypertrophy and right-sided heart failure has been the cause of death.

Cause of right ventricular hypertrophy in 88 cases out of 2,000 post-mortems (22.11.50–8.8.56).

| | |
|---|---|
| Chronic lung infection, emphysema or pneumokoniosis | 55 |
| Rheumatic valvular disease (mitral stenosis) | 13 |
| Congenital heart disease | 8 |
| Following left ventricular failure due to hypertension, calcific aortic disease or coronary infarction | 6 |
| Severe kyphoscoliosis | 3 |
| Multiple pulmonary embolism | 1 |
| Muscular dystrophy | 1 |
| Tumour of left atrium (pseudo-myxoma) | 1 |

This, therefore, was a man with kyphoscoliosis, the cause of which is not apparent. There is quite a large group in which there is no obvious cause and these are often described as postural. He developed right ventricular hypertrophy without any gross intrinsic lung disease and he suddenly went into failure and died.

*Professor Perry:* I wonder if Dr. Johnson could tell us how kinked the aorta was and whether there was any fibrosis around the root of the pulmonary artery as described in one of Coombs' cases.

*Dr. Johnson:* I was very struck by the lack of any obvious mechanical factor which could have given rise to the pulmonary hypertension. I am convinced that there was no serious kinking of the aorta; if there had been aortic obstruction one would expect left-sided cardiac hypertrophy. It is difficult to believe that there might have been any kinking of the pulmonary veins; if this had been present one would expect chronic pulmonary congestion. The lungs were normal apart from the changes of pulmonary hypertension.

*Dr. Sutton:* What about the pulmonary arterioles?

*Dr. Johnson:* The degree of arteriolar hypertrophy is difficult to assess exactly. They are thick-walled.

*Dr. Sutton:* Is there any question of devascularisation at capillary level?

*Dr. Johnson:* There was no evidence of endarteritis or any destruction of the capillary bed.

There is a perfectly good hypothesis to account for this condition. In normal people on exercise there is a fall in the diastolic pressure in the pulmonary circulation so that in actual fact the work done by the right heart, which determines the mass of the muscle in it, changes negligibly. If, on the other hand, you take people with any of these lung diseases, pulmonary emphysema, for example, and give them increased work, the pressure, both diastolic and systolic, rises and the work of the right heart increases. Whereas in normal people the increase may be only 10 per cent, in people with emphysema the pulmonary pressure may double or even treble. This may tie in with the degree of oxygenation of the blood. In the normal if the concentration of oxygen in inspired air is reduced to half there is a very considerable rise in the pulmonary arterial pressure, and it appears possible that in this man with poor lung movements because of his kyphoscoliosis, deficient oxygenation of the blood leads to depression of the normal reflexes which should result in dilatation of the pulmonary circulation during exercise. This seems to be the first stage. Vascular changes appear and a vicious circle occurs until there is increased pulmonary arterial pressure even at rest.

*Professor Perry:* I think your theory may be right but I would put it the other way: that deficient oxygenation of the blood and chronic anoxia may give rise to increased tone in the pulmonary arterioles—pulmonary hypertension. I am still puzzled why these people get rapidly increasing dyspnoea first and yet on post-mortem you say this man's lungs were dry.

*Dr. Johnson:* Their dyspnoea is due to decreased vital capacity.

*Professor Perry:* Then why does the dyspnoea suddenly increase rapidly in intensity? Was there any evidence of recent chest infection?

*Dr. Johnson:* These lungs are quite normal lungs. There is no fibrosis nor evidence of any recent pulmonary infection.

*Professor Hewer:* Dr. Johnson showed us a section of the vertebra with the kink and there is some sclerosis of the bone and nothing else to explain the kyphoscoliosis at all. I have always thought the postural hypothesis for kyphoscoliosis is a very poor argument.

I have brought from the museum a specimen of the whole trachea of a man of 20 who was a hunchback as a result of Pott's disease. He had tuberculous caries of the spine when he was six and died after about 48 hours of influenza during an outbreak in 1940. He had been very subject to coughs and colds and was absolutely blue. The length of his trachea was only 9 cm. and this is an indication of the degree of deformity in his case. He died of asphyxia because of accumulation of mucus in his bronchi and pulmonary collapse.

I suggest that the chief problem needing investigation is the true mechanism of production of kyphoscoliosis in childhood in the absence of Pott's disease or poliomyelitis. I find it hard to believe that adopting a bad posture could possibly cause it.

*Professor Perry:* I do agree with you about wanting to know the cause of kyphoscoliosis. In fact, Coombs justified his publishing the record of his four cases in the

*British Journal of Surgery* as follows: "Whatever the explanation it is clear that there is a real danger of untimely death from cardiac failure in the victims of angular deformity of the spine, and it is by way of affording to the orthopaedic surgeon an added reason for attempting to prevent that deformity that this note was written."

*Professor Hewer:* I think Dr. Coombs reported two cases in one family with idiopathic kyphoscoliosis. Were both of these attributed to faulty posture?

*Professor Perry:* Yes, Case 4 whose brother had been similarly deformed and died of heart failure. There was no post-mortem but there was no known cause for the kyphoscoliosis.

I do not believe that spinal deformity is as common as it used to be. Has the medical inspection of schoolchildren and so-called remedial exercises done any good?

*Professor Neale:* Not a bit.

*Dr. Sutton:* These so-called postural deformities occur in children with malnutrition. They are sub-rachitic.

*Professor Neale:* If you put them in a medical support or even a metal cage they will still develop kyphoscoliosis and bend the steel. (Laughter.)

*Dr. Gaskell.* With regard to the spine I would like to enquire from Dr. Johnson whether there was a primary cervical curve above the dorsal kyphosis?

At Frenchay Hospital we have quite frequently seen cervical cord or nerve-root compression developing in early middle-age where there was a primary cervical anomaly of Klippel-Feil type (and secondary dorsal kyphoscoliosis). Such cases are notorious "anaesthetic risks" due both to the rigid thorax and reduced phrenic innervation. These factors could be operative in the case under discussion.

*Dr. Johnson:* There was a curve in the cervical region, but not more than we would have expected. There was no defect at the base of the skull, nor obvious cord compression in the cervical area.

(*Addendum by Dr. Johnson:* One feature of this case which escaped discussion during the conference was the coincidental gastric ulceration. The incidence of subacute or chronic gastric ulceration in the 88 cases of pulmonary hypertension in the above table is 11·4 per cent (ten cases) and of acute ulceration 5·7 per cent (5 cases). This compares with 3·8 per cent, 0·7 per cent and 1·8 per cent for chronic subacute and acute gastric ulceration respectively in the whole series of 2,000 autopsies. There is no evidence of increased incidence of gastric ulceration, other than erosions, in passive systemic congestion from the figures of the extensive German literature and the possible neuro-vascular background to both pulmonary hypertension and gastric ulceration opens an interesting line of speculation.)

### REFERENCES

Coombs, Carey F. (1930). *Brit. J. Surg.*, **18**, 326–328.

