# Apology

**Published:** 1958-04

**Authors:** 


### *An Apology to Dr. G. E. F. Sutton*

Dr. Sutton points out that at the Clinico-Pathological Conference published in the January number of this *Journal* he is quoted as saying "These so-called postural deformities occur in children with malnutrition. They are sub-rachitic". Yet what he actually said was that in former days the mild cases of postural deformity were often sub-rachitic.

We apologise to Dr. Sutton for reporting him incorrectly, and for omitting to send the text to him for correction before it was published.

*Editor.*

